# Interactive webcam travel: supporting wildlife tourism and conservation during COVID-19 lockdowns

**DOI:** 10.1007/s40558-023-00242-3

**Published:** 2023-02-13

**Authors:** Madelene Blaer

**Affiliations:** Victoria University, 372-378 Little Lonsdale St, Melbourne, VIC 3000 Australia

**Keywords:** Webcams, Livestreaming, Virtual tourism, Wildlife tourism, Conservation

## Abstract

Using the conceptual frameworks and theories of virtual tourism, telepresence and para-social interactions, this exploratory study investigates an innovative campaign employed by a nature-based wildlife tourism operator as a response to the COVID-19 lockdowns and travel restrictions of 2020/21. Insights are provided into a unique model of webcam livestreaming that is scheduled, hosted and interactive. Over 73,000 social media comments and 590 survey responses from webcam viewers were analysed and indicate that watching the livestream had positive impacts for tourism recovery and conservation action. Research findings suggest that interactive webcam travel can affect travel behaviour and conservation awareness and action in part through building and engaging online communities and supporting a sense of connection with nature. This study contributes new knowledge to the emerging research on webcam livestreaming in tourism. As a subset of virtual tourism, interactive webcam travel emerges as an alternative to more costly forms of virtual reality for industry practitioners and stakeholders to engage new and old audiences, especially in the context of tourism recovery initiatives after disasters and crises that prevent or limit physical visitation.

## Introduction

‘Aussie Penguin Parade is lockdown live-stream hit’ (Global Times 2020 August 26).

‘Livestream of Australia’s Penguin Parade brings smiles around the world’ (Global Voices 2020 August 30).

‘Phillip Island’s Penguin Parade huge success with eight million viewers’ (7 News 2020 September 13).

The sample of news headlines above illustrate the impact generated by the virtualisation of a popular travel experience during the COVID-19 lockdowns and travel restrictions of 2020/21. Pre-pandemic, the last two decades saw considerable uptake of virtual experiences in the tourism industry, particularly as a destination marketing tool. Virtual reality provides marketers opportunities to offer people more compelling imagery of tourism products, giving them a sense of what it is like to be there and assists with the ‘try before you buy’ issue in travel (Tussyadiah et al. 2017). Studies provide empirical evidence confirming the effectiveness of virtual reality in shaping consumers’ attitude and behaviour in terms of destination preference and increasing visitation intention (Tussyadiah et al. 2018; Lo and Cheng 2020; Wu and Lai 2022).

During the pandemic, webcam livestreaming viewership numbers dramatically increased. Hampshire Zoo in the UK reported that viewers reached 64 thousand while in lockdown, a 27 thousand per cent increase from the week before (Hirons 2020) and monthly webcam views for Edinburgh Zoo rose from 100 thousand to more than 5 million (Beddington 2020). While predominantly nature-based, the featured destinations and attractions varied, including city centres, holiday resorts, beaches, zoos, aquariums and wildlife sites (Bogle 2020). These viewer numbers are important for tourism operators during states of emergency in various ways. This includes increasing general awareness, maintaining communication with stakeholders throughout the crisis, as well as in the recovery stage, when businesses are looking to attract and welcome visitors back. As a result, there is increased opportunity and interest for the use of immersive technologies in tourism in a post-COVID landscape (Yung et al. 2021).

Phillip Island Nature Parks (PINP) in Victoria, Australia, was one of the organisations to offer virtual travel during the pandemic, live broadcasting their iconic Penguin Parade via webcams. The online event attracted global media attention and viewership reports of 25 million by its final nightly stream (Wilkinson 2020). Known as Live Penguin TV, this virtual travel experience was different in several ways to the usual place-based live webcam:

1. It was scheduled to air nightly at sundown for about an hour, rather than being available 24/7
2. Park ranger hosts provided live expert commentary and on occasion featured guest presenters to discuss conservation issues
3. Multiple cameras provided a range of perspectives, controlled by the hosts who could respond in real-time and zoom in on live events
4. Online audiences could ask questions and interact with hosts and other viewers via social media chat functions

While studies show that online and virtual reality experiences can reinforce travel motivation and conservation actions (Govers and Go 2006; Jansson 2002; Hofman et al. 2021) and therefore assist with the recovery of the tourism industry (Lu et al. 2022), there is a paucity of research that examines the use of live webcams in this regard. Earlier research has also established that nature-based and wildlife experiences can prompt people to adopt conservation behaviours (Ballantyne et al. 2009, 2011; Higginbottom and Tribe 2004), yet the ability of virtualised wildlife and nature-based experiences to do so is less clear. This exploratory study intends to help fill these gaps and contribute to this emerging area of research by analysing an innovative approach to webcam livestreaming by a nature-based wildlife tourism operator. In particular, the research aim is to examine how interactive webcam travel experiences can affect viewers’ attitudes and behaviours in terms of travel and conservation awareness and action.

The structure of this paper consists of, firstly, a review of the literature and research pertaining to virtual tourism and webcam experiences, before outlining the methodological approach. A brief history to PINP and the pandemic’s impact on the Victorian visitor economy is then provided. The research findings are presented with the primary data accompanied by a discussion of how it relates to the existing literature. The paper concludes by outlining the potential implications, contribution and usefulness of the research findings for knowledge building and industry practice, as well as the study’s limitations and suggestions for future research.

## Webcam travel, virtual tourism and livestreaming

The term webcam travel was introduced as a concept in the academic literature only recently, defined as visiting place-based webcams online (Jarratt 2020a). Conversely, virtual tourism is a highly generalised and commonly used term. While there is no universally accepted definition, a usual understanding is that virtual tourism is based upon the concept of telepresence, which is understood as media users’ psychological state in feeling lost or immersed in the mediated environment or the degree to which users feel that they are somewhere other than their actual environment (Steur 1992). Studies have found that a higher level of telepresence can elicit stronger (re)visit intentions (Ying et al. 2021). Using VR 360° video technology, Lo and Cheng (2020) found that viewers perceived an advertised hotel more favourably and showed a greater intention to book rooms there when they experienced a more intense sense of presence.

Virtual reality has received significant attention in tourism studies and is often inseparably linked with virtual tourism. Defined as a simulation or representation of a particular environment using media, virtual reality is often seen to require wearable technology, but Steur (1992) explains that it is possible to define virtual reality without reference to hardware. A myriad of products exists in the marketplace, ranging from basic novelty type applications (e.g. Google cardboard) through to advanced cutting-edge technologies (e.g. Oculus Rift headset, haptic sensors). It is also possible to engage in partially immersive experiences on flat screen devices (e.g. 360-degree video, computer games like Second Life). A review of post-millennium publications identified 1049 articles on virtual reality in tourism, which steadily increased over the years (Moro et al. 2019). Such a review of publications on webcams in tourism does not appear to exist, most likely due to the dearth of scientific papers on the subject.

Despite receiving limited attention by tourism scholars, webcams are not a new phenomenon and are commonly used in the tourism industry. Topics that have been explored include the use of webcam images as a data source (Timothy and Groves 2001), to monitor tourist behaviour (Ibarra 2011), and more recently, a comparison of real and webcam wildlife experiences ability to produce desired awareness and action responses (Skibins and Sharp 2019). One empirical study of webcam travel experiences during the COVID-19 pandemic suggested that people were using webcams more frequently to elicit feelings of freedom, a nostalgia for pre-COVID times and a sense of connection with place (Jarratt 2020b). Webcam travel has also recently been linked to generating psychological well-being amongst online viewers (Lee et al 2022).

Webcams have evolved alongside technological innovations to become the livestreamed webcasts known today that provide an online portal to the material world through unedited live place-based footage. Jarratt (2020a) supports the argument that webcam travel does not easily fit many definitions of virtual tourism, finding most are too narrow and biased towards virtual reality. He put forward that definitions of virtual tourism need to be expanded to accommodate ‘other technologies that can only offer a portal to a ‘real’ place, as opposed to one which is amended, created or recreated’ (Jarratt 2020a, 2). In this way, webcams are differentiated from other virtual tourism technologies, such as virtual reality, that can create and simulate impossible experiences, such as deep space travel or walking with dinosaurs. Rather, webcams are purely linked to physical places in the material world. He further differentiates webcams in that they ‘are not ‘thinking’, they do not offer a perspective on the past and they do not necessarily need interpretation of any kind. Nor is there a performance, for the subjects of these cameras are usually oblivious to their presence’ (Jarratt 2020a, 2). This current study adopts the all-inclusive classification of virtual tourism and sees webcam travel as a sub-category, ‘made distinct by its live nature and relatively simple technology’ (Jarratt 2020a, 2).

While linked to webcams, livestreaming is not device specific. For example, where a stationary webcam can livestream a specific event or scene, a moveable hand-held device can livestream the view captured as it travels around. It is this mobile form of livestreaming that features predominantly in the literature. The tourist as live streamer/broadcaster concept has been examined by Deng et al. (2019, 214), who proposed that livestreaming tourism can be defined as a ‘simultaneous, immediate, social and vicarious form of sociotechnical tourism’ characterised by real time visual production and consumption of blended experiences. Comparing how tourism visual mediums differ, they argued that livestreaming allows destinations and experiences to be created, consumed and shared in ways that are fundamentally different in seven ways, including low/medium versus high presence, pre-recorded versus real-time, virtual versus blended space, raw/edited versus unedited performance, contrived versus staged authenticity, static versus spontaneous story-telling, as well as asynchronous versus synchronous social interactions. The social dimension does not necessarily fit with the standard webcam, but it could apply to an interactive model.

From a human behaviour perspective, Hu et al. (2017) examined why audiences choose to keep watching livestreaming video platforms and found that para-social interaction and self-congruity can affect viewers’ engagement. The theory of para-social interaction, proposed by Horton and Wohl (1956), describes a sense of mutual awareness, closeness and intimacy with media personas (e.g. TV news hosts, celebrities, influencers). Despite being physically distanced from such characters, viewers gain increasing personal attachment, relationship investment and loyalty towards them. Theories of social interaction present an opportunity for studying the social dimensions of livestreaming tourism (Deng et al. 2019), including livestreamed webcams, which could trigger para-social interactions through enhanced interactivity elements provided by social media, such as audiences commenting and communicating with the presenter as well as each other via text-based chat room functions.

To conclude this literature review section, the theories of telepresence and para-social interaction do not appear to have been examined in the context of virtual webcam travel amidst global travel restrictions. Extant studies show that virtual tourism experiences can reinforce travel motivation and encourage conservation action; there is a knowledge gap in the research, however, that explores the use of livestreamed webcams in this regard. This study therefore intends to contribute to the emerging research on webcam travel by focussing on interactive experiences afforded through Live Penguin TV.

## Research methods

The research utilised a mixed-method approach, which was deemed most suitable given the exploratory nature of the study. Exploratory studies are often employed to examine a new research theme or to address an existing issue from a new perspective (Mason et al 2009), such as in the case of webcam travel. The combination of qualitative and quantitative data can contribute to a better understanding of a research problem (Creswell 2015) and compensates for the disadvantages of each individual method (Khoo-Lattimore et al 2019), whereby data triangulation through mixed-methods minimises the limitations (Teddlie and Tashakkori 2009). In particular, the sequential mixed-method (Mason et al 2009) was used in this research, which fits within Creswell’s (2009) sequential exploratory strategy, where the first stage of research involves qualitative data collection and analysis that is used to inform the second quantitative stage that builds on the first stage. An etic epistemology was adopted in that the researcher’s situatedness was passive (e.g. the researcher watched several livestreams but did not participate or interact with the presenters or other viewers).

Data were primarily generated by an online survey and social media sources, supported by online correspondence and meetings with PINP staff, an online in-depth interview with management and desktop research. Firstly, correspondence by virtual meetings and email with PINP management and staff provided the researcher with an understanding of the background to Live Penguin TV. An hour-long in-depth interview was held with the marketing manager and recorded to explore the strategic thinking behind the campaign. This provided the opportunity to delve deeper into issues highlighted in the media in more detail. This manager was purposively selected to interview as they were deemed to have the experience and knowledge required to provide the information needed to achieve the research objectives. While the in-depth interview was held online due to travel restrictions, limiting the ability to read body language, the interviewer established rapport and trust through frequent email communication and the interview generated rich data. The interviewee was generous with their time and the length of the interview was determined by a point of data saturation, where no new themes emerged from the questioning (Tracy 2010).

The survey was developed and distributed using Qualtrics, an online survey building and analysis tool. The survey was available from 4 December 2020 to 7 January 2021 and generated 611 responses; 21 incomplete responses were excluded from the analysis, resulting in a total of 590 usable responses. The questionnaire took 5–8 min for respondents to complete and consisted of 20 total items (seven demographic items and 13 substantive survey items), all of which were germane to the study. Most questions provided quantitative data and included pre-coded response sets. Open-ended questions captured qualitative sentiments that could be triangulated with social media data (Teddlie and Tashakkori 2009).

**Fig. 1:**
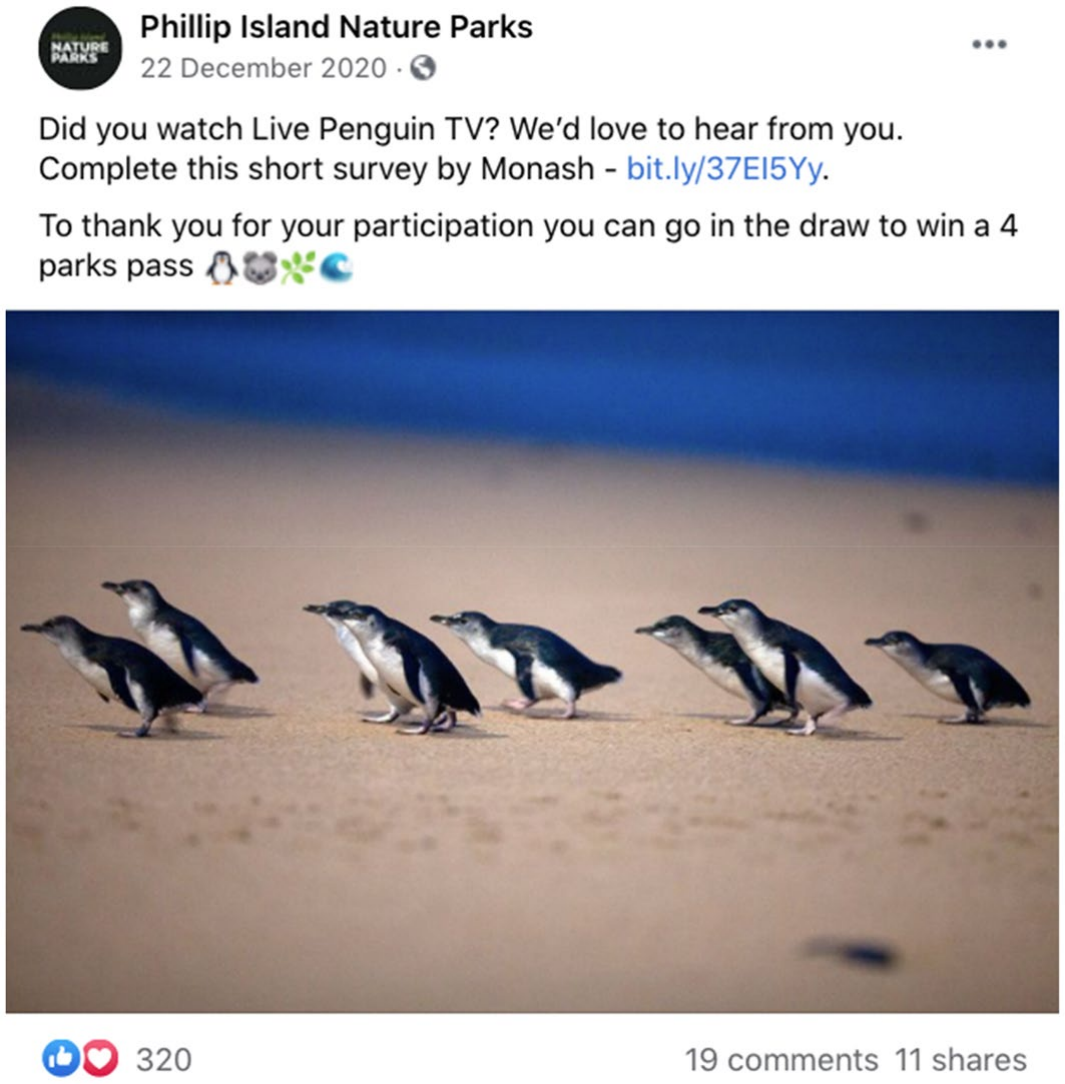
Survey link on Phillip Island PINP’s Facebook post with incentive to participate

**Fig. 2:**
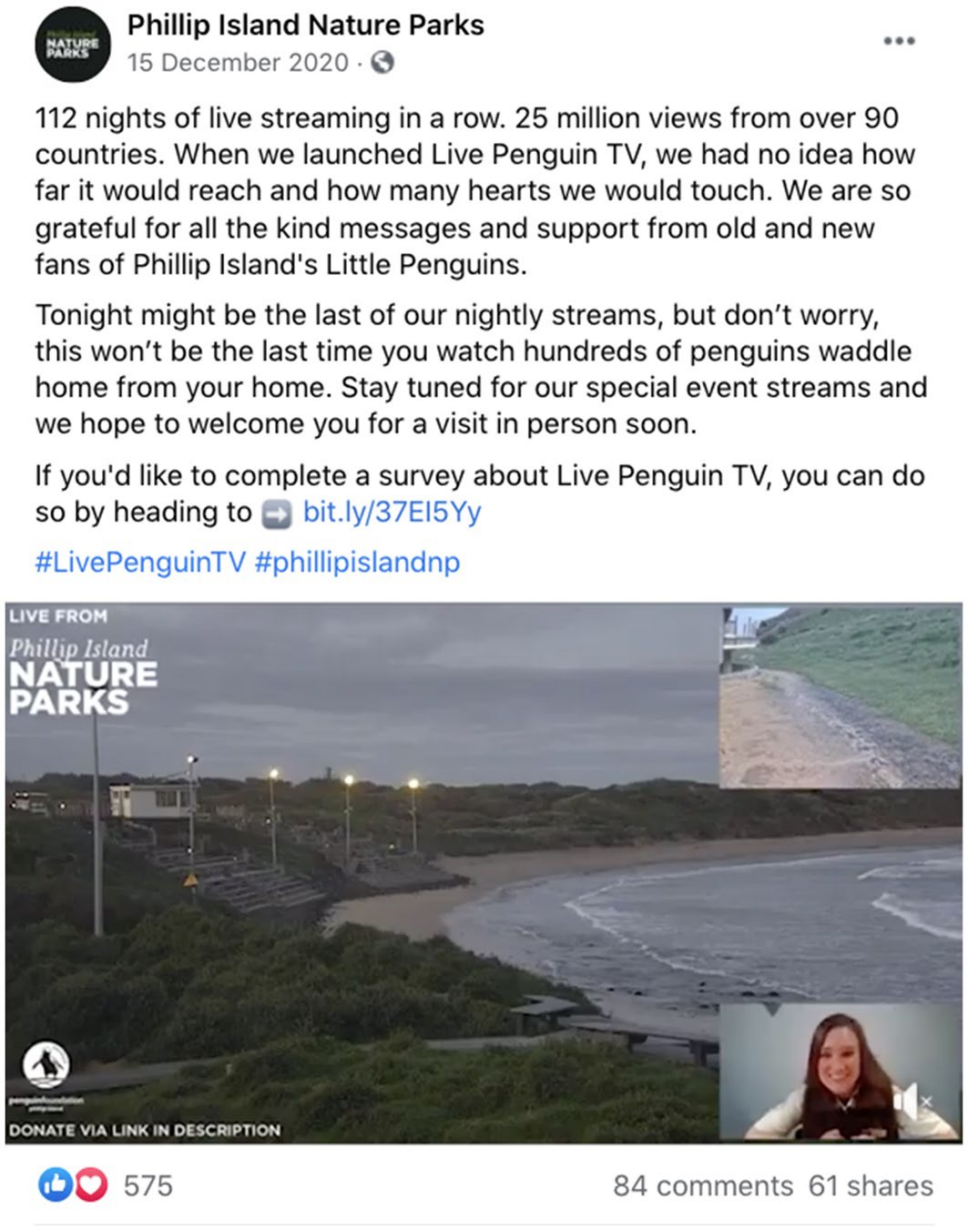
Social media call for survey respondents

The questionnaire was shared and promoted on PINP’s website and social media accounts. Links to the survey were positioned prominently using pinned posts about Live Penguin TV on Facebook and YouTube, as well as mentioned in two specific posts on Facebook, namely a highlights video reel and a dedicated post specifically about the survey with an incentive to participate (see Figs. 1 and 2). Recruiting respondents on social media greatly expands the reach of a study in a very cost-effective manner (Quinn 2020) and these social media channels were deemed highly appropriate as this is where viewers accessed Live Penguin TV, however, the sample was generated using convenience methods and hence is not necessarily representative of the broader population.

Social media data were downloaded by PINP for the Facebook and YouTube accounts after the final livestream in mid-December 2020 and provided to the researcher who analysed the key metrics for 114 YouTube video posts, 111 Facebook Live video posts and 23 Facebook posts about Live Penguin TV (photo, shared video, link or status update), posted between 24 August and 16 December 2020. Users’ online interactions are geo-localised and leave a ‘digital footprint’ of where and when the interaction took place (Flores-Ruiz et al. 2021), providing useful insights into the geographical reach of the livestreams and users’ activity (e.g. likes, comments, shares). Social media comments are publicly available data and software was used to retrieve and export 62,495 YouTube and 10,780 Facebook viewer comments for analysis. The discrepancy in the number of video posts and comments between platforms was explained by PINP’s social media manager who clarified that the streaming software did not always connect correctly with Facebook. This meant that only 16 Facebook videos captured comments.

Together, this large volume of data was analysed to generate knowledge and provided a more holistic, insightful overview of virtual tourists’ attitudes and activity (Flores-Ruiz et al. 2021). The quality of social media data, however, has been questioned in terms of reliability, validity and generalizability of research findings with issues raised including platform biases, data availability biases and data authenticity issues (Xiang 2018). The validity of online survey data has been similarly questioned, although it has been found that neither pure paper-based or online surveys are unbiased, and no differences exist in the contamination of data by response styles; benefits of online surveys include a lower dropout rate and less incomplete data (Dolnicar et al. 2009). It is therefore acknowledged that the samples drawn are not representative of the broader population and are limited to a sample of self-selected Facebook and YouTube users, which are the social media platforms on which the webcast was streamed.

Data analysis was performed both during data generation, mainly during the desktop research and interview as themes were noted by the researcher, and post data collection, mainly for the survey and social media analysis, following the sequential mixed-method (Mason et al. 2009). While Qualtrics was used for quantitative data analysis and to generate data visualisations for the survey results, Excel was used for the social media data to calculate descriptive statistics. For the sentiment analysis, comments were often brief, hence themes were easily identified through keyword filters and sorting.

## Brief background to the Penguin Parade

Established in 1996 by the State Government of Victoria, PINP is a not-for-profit organisation that manages 1,805 hectares of Crown Land. This land is part of the UNESCO Western Port Biosphere Reserve and encompasses wildlife sanctuaries, coastlines and internationally protected wetlands. Located around 90 min’ drive south of Melbourne, PINP is the largest employer for the region and the Penguin Parade is an internationally recognised attraction. Each night at sunset, penguins group together at sea in the hundreds or thousands, in preparation to waddle across the beach and return to their burrows in the sand dunes.

The first visitor centre was constructed in the 1960s and has undergone several redevelopments, most recently in 2019 with a new $58.2 million award-winning visitor centre as one part of a strategic master plan to better protect the penguins and their environment. Relocating the site of the visitor centre meant that significant penguin habitat was restored and the modern building was designed to be environmentally sustainable and manage visitor numbers, which reached 4500 nightly pre-pandemic. PINP is the most popular wildlife tourist destination in the state of Victoria, drawing 740,000 visitors in 2018 (Rodell 2019).

PINP engages in a number of protective and conservation measures through research, education programs, habitat restoration and management, wildlife rehabilitation and predator control programs. Operations are sustained by visitation and further funding is attracted through the Penguin Foundation, which allows people to donate or symbolically adopt a wild animal on Phillip Island. These programs and conservation initiatives gain added visibility through tourism and faced unprecedented challenges due to COVID-19 as PINP was entirely self-funded though their visitor programs, tourism experiences and donations.

Tourism related businesses were forced to close during the Victorian lockdowns. Located outside the boundaries of metropolitan Melbourne, PINP was able to reopen when restrictions eased in regional Victoria, yet significant challenges remained. Unlike some other destinations that turned to domestic tourism, PINP has faced challenges with state borders closed and Melbourne’s 5 million residents in lockdown, in effect limiting the market for the most part to regional Victoria since the start of the pandemic. As one of Victoria’s key tourism destinations, Phillip Island was identified as being dependent on tourism (Austrade 2020) and significantly affected by the lack of visitors caused by the pandemic. Recent studies have addressed the pandemic’s implications for wildlife tourism, noting that the drastic decline in visitation and revenue has led to either a severe reduction or total halt in conservation efforts (Kideghesho et al. 2021; Newsome 2020).

## Findings and discussion

The research findings are presented with the triangulated sources of primary data from the interview, online survey and social media data integrated with a discussion of how it relates to the existing literature, as is common practice in qualitative research (Jones et al. 2013) to form a rich and cohesively narrated findings section. The conceptualisation of Live Penguin TV coincided with the first lockdown in Victoria from mid-March to the end of May 2020. The marketing manager had noticed the rise in webcam viewership around the world and observed that fixed cameras did not always provide engaging footage, for example, if zoo animals were out of shot. With businesses required to shut down for indefinite periods, tourism operators were fearful of losing their relevance with their markets. In the case of PINP, which is dependent on visitor revenue, this connection was crucial.

… we have to get out there in the public, even if we’re closed, so that people still know we’re doing great conservation work. But how do we actually show people what we’re doing? That’s when I thought we could have cameras at the Penguin Parade. We were running out of money while we were closed and I wanted the government to understand what we were doing. I think the fact that we had prominence really helped us. I know that we got the [government support] funding and a lot of that was to do with people who saw the penguin livestreaming *(Marketing manager, Interview).*

Live Penguin TV hence served several purposes, including maintaining a connection and relevance with audiences, securing government funding to continue critical conservation operations, as well as retaining as many staff as possible during the lockdowns.

We had government funding, so we could keep the current workforce, so probably 60 or 70 percent of our staff in the [visitor experience] team, ended up doing conservation work, like planting trees. *(Marketing manager, Interview).*

Putting the idea into action proved challenging though; government approval had to be sought and requirements met, such as a tender process for the cameras and permits to set up the hardware during the lockdowns. This did, however, provide time to prepare a makeshift studio and select and prepare staff, including rehearsals with the park rangers who would eventually become the ‘faces’ of Live Penguin TV. Melbourne entered a second lockdown with stricter controls when Live Penguin TV was ready in August 2020 and this likely played into its viewership numbers because Melbournians were unable travel further than a 5 km radius. Combined with a curfew and outdoor time-limit of one hour, these virus containment measures made daytrips impossible. There was still trepidation from management about how Live Penguin TV would perform and significant efforts were put into promoting the campaign.

I wasn’t sure if anyone would watch … or if the cameras would freeze, a penguin could die on screen, the talent won’t turn up, someone will hack it … it was a lot of pressure. But then we got our PR agency to promote it, so we had a campaign prior to launching, which was picked up by all mainstream media … If I’d launched it in March or April, it wouldn’t have the same impact. August was a pretty dark time and Live Penguin TV was one of the only good news stories out there *(Marketing manager, Interview).*

In-kind industry support from state and national tourism organisations also generated awareness, as the marketing manager explained: ‘they got behind it by cross-posting. We can feature it on our Facebook page, but if they repost, the numbers triple, Visit Victoria and Tourism Australia did a lot of promotion overseas for us’. PINP’s own social media following on Facebook and YouTube quadrupled during the campaign, increasing the effectiveness of marketing on their own channels. The campaign generated international media attention and a Google search lists news articles from China, India, Indonesia, France, Netherlands, Pakistan, Singapore, South America, South Africa and the United Kingdom.

Social media data confirmed the news media reports regarding viewership numbers, with the top performing livestream on the launch night reaching 840 thousand unique views. Nightly viewership numbers then averaged out to about 25 thousand, which is a significant sustained engagement over the 112 consecutive days that the livestream aired, with more than 7.4 million unique users viewing during that time. Viewers were highly engaged on social media, resulting in thousands of comments and questions nightly. Chat comments and questions averaged 600 a night on each platform but increased up to 5000 on three specific nights around the launch.

Some viewers asked the same questions repeatedly each night – one viewer reposted the same question 100 times on one night—because their post may have become lost in the rapid feed of comments and/or unanswered by the hosts. Other frustrated viewers referred to this as ‘spamming’ in the chat feed and would report users to have their accounts blocked from accessing the site. Other unanticipated challenges arose, including fraudulent social media accounts posing as being associated with PINP and trying to charge viewers for access to the livestream. Additional ranger staff were brought into the project to help answer questions and communicate with the online audience, as this proved the most effective way to manage expectations.

### Audience profile

Social media data revealed that Live Penguin TV was viewed from 119 countries across the world. The majority of survey respondents were located in Australia, with more than half (66%) located in Victoria. Within this state, more than half (62%) were watching from Melbourne. Interstate respondents within Australia accounted for 22 per cent, mainly in New South Wales (11%) and Queensland (8%). International respondents accounted for 12 per cent, mainly from the United Kingdom. Table 1 shows the geographical locations of survey respondents.

**Table 1:** Geographical location of survey respondents

	Count	Percentage
*Survey respondent geographical location*		
Intrastate (Victoria)	351	66%
Melbourne	219	–
Regional	132	–
Interstate	115	22%
New South Wales	49	–
Queensland	36	–
South Australia	11	–
Tasmania	8	–
Western Australia	5	–
Northern Territory	3	–
Australian Capital Territory	3	–
International	67	12%
United Kingdom	24	–
United States of America	10	–
Singapore	6	–
China	4	–
Hong Kong	4	–
New Zealand	4	–
Germany	3	–
Japan	3	–
Canada	2	–
Austria	1	–
Italy	1	–
Malaysia	1	–
Malta	1	–
Philippines	1	–
Portugal	1	–
United Arab Emirates	1	–

The relatively small number of respondents from China and absence of survey respondents from India, Indonesia and Korea were noted as these are strong market segments for the Penguin Parade. International internet censorship might explain some of these low levels of viewership. Another partial explanation for the geographical profile of respondents is that the offer used to incentivise participation was the chance to win two visitor passes, which was likely to be mostly attractive to viewers in Victoria, considering travel restrictions.

The majority (almost 90%) of respondents were female and 67% were aged between 40 and 69, mostly employed full time (36%) or retired (22%). The gender profile of viewers on Facebook showed 72% female and 28% male and the average age of viewers on Facebook (35–44) was younger than survey respondents.

Live Penguin TV became a ritual for viewers during the lockdown in Victoria, as shown by survey respondent viewing behaviour in Table 2. There was a cohort of regular viewers with 28% watching on a nightly basis and another 38% watching at least once weekly. Almost half of the survey respondents watched by themselves and another 48% watched with their family (partner, relatives, children), which can be linked to most viewers being in lockdown with their household.

**Table 2:** Survey respondents webcam viewing behaviour

	Count	Percentage
How often respondents watched Live Penguin TV		
One time	13	2%
Two times	18	18%
Three to four times	71	12%
Five to ten times	102	17%
Once a week	28	5%
Two to three times a week	81	14%
Four to six times a week	113	19%
Daily	164	28%
Who respondents watched Live Penguin TV with		
By myself	364	49%
With partner	134	18%
With family/relatives	113	15%
With children	116	15%
With friend/s	16	2%
With colleague/s	5	1%

### Presence and connection

Survey respondents reported that the top reasons for watching Live Penguin TV related to the conservation of wildlife and the natural world, education about it and feeling connected. Respondents indicated that seeing animals in their natural habitat (83%), learning more about penguins (73%) learning about conservation (60%) and feeling connected with the place/ outside world (60%) were important factors in their decision to watch the livestream. These reasons were selected over being entertained and simply looking for something to do while in lockdown.

The extended comments provided by survey respondents and social media data revealed other major themes, including Live Penguin TV’s role in helping viewers cope with lockdown isolation, especially for those suffering from extreme loneliness, depression and anxiety due to the pandemic. The following examples demonstrate this sub-theme:

It kept me sane during lockdown as I had something special to look forward to nightly (*Survey respondent, Melbourne, female, 50–59, watched daily*).

It helped me so so much with my mental health by distracting me (*Survey respondent, South Australia, female, 50–59, watched 4–6 times weekly*).

This brilliant live penguin got me through deep depression of solo isolation by giving me a chance to see close up these magical creatures (*Survey respondent, Gippsland in Victoria, female, 70–79, watched 4–6 times weekly*).

This was part of my mindfulness activities that I practiced to keep myself sane during the stressful lockdown period (*Survey respondent, Melbourne, male, 40–49, watched 4–6 times weekly*).

You guys have helped get us through stage 4 lockdown @Phillip Island Nature Parks so huge thanks huck huck huck *(YouTube user)*

I find it difficult to explain how important this stream has been to my survival. The stress of the lockdown with my sick husband who then died. The total isolation, then the death of another family member... The wonderful penguins and their passionate rangers became a very real connection. Thank you But for you I may not have survived *(Facebook user).*

For some, physical travel was not possible for a variety of reasons and the interactive webcam livestream provided a vicarious experience through telepresence. The results support those of Hofman et al. (2021), where study participants felt a sense of connectedness to nature as they were transported to the destination. Teletransportation is illustrated in the following quotes:

They made you feel that you were really there (*Survey respondent, Melbourne, female, 50–59, watched 2–3 times weekly)*.

I'm housebound, so traveling via the Internet is a treat. I may never go in person, but I can go in my mind (*Survey respondent, Queensland, female, 50–59, watched 2–3 times weekly*).

It gives people a chance to experience the real thing in preparation of visit in real life (*Survey respondent, Melbourne, female, 60–69, watched 2 times*).

Jarratt’s (2020b) findings are also reinforced with respondents indicating that the webcam livestream provided a sense of connection with the outside world, especially nature and wildlife, as well as a reminiscence of pre-COVID times with people reflecting on past travels. Beyond this though, the most prominent subject was that a sense of community was generated with other viewers. All of these factors supported viewers’ wellbeing, supporting the suggestion that webcam travel can contribute to psychological wellbeing amongst online viewers (Lee et al 2022). The examples provided below reflect these themes:

It’s been great to feel a part of nature (*Survey respondent, Victoria, male, 30–39, watched 4–6 times weekly*).

Great way to connect with the natural world and other penguin fans (*Survey respondent, UK, female, 50-59, watched 4–6 times weekly*).

It was a great experience and really helped us feel connected to the outside world even when we couldn’t be (*Survey respondent, Victoria, female, 40–49, watched 2–3 times weekly*).

Feels nice to be a part of a virtual community (*Survey respondent, Melbourne, female, 40–49, watched daily*).

Prompted many memories of family holidays on Phillip Island (*Survey respondent, Melbourne, female, 50–59, watched 5–10 times*).

I’m in Lincolnshire, UK. Visited you in 1999 (*Facebook user*).

It's honestly been quite a difficult year for me and you gave me something happy to look forward to every night. We became a Waddle Watchers family and I hope that we will still huk huk out to each other and raft up for future Livestreams (*Facebook user*).

In this way, Live Penguin TV generated a sense of community and connection with other online users. Some viewers collectively referred to themselves as ‘Waddle Watchers’, and after the final nightly livestream aired, they established a private Facebook Group for interested viewers to keep in touch. The group was created on 7 December 2020 and is still highly active with 1068 posts in the month of February 2021. Engagement was also generated through an element of gamification, whereby viewers would guess how many penguins had arrived the previous night. The regular park ranger hosts, who became like celebrities, were highly valued by viewers, who appeared to develop a para-social relationship (Horton and Wohl 1956). Viewers reported wanting to visit the Penguin Parade in order to meet and thank the hosts. The sense of community with other online viewers and closeness with the hosts are reflected in the following sample of quotes:

Loved the entertaining Rangers/Counters and also the live chat function on You Tube and Facebook Live - felt connected with others worldwide (*Survey respondent, New South Wales, female, 50–59, watched 5–10 times*).

It was fun to engage with the Rangers & the regular ‘waddle watchers’ (*Survey respondent, Melbourne, female, 60–69, watched daily*).

It became like family (*Survey respondent, Mornington Peninsula in Victoria, female, 50–59, watched 4–6 times weekly*).

Bonding with other fellow waddler watchers (*Survey respondent, Singapore, male, 40–49, watched daily*).

You are very knowledgeable and when I come for a visit I will look out for you (*YouTube user*).

… it has have been an absolute pleasure watching you in the lockdown you are so friendly and out going. You are in the perfect job for your personality. I look forward to meeting you in person (*YouTube user*).

It will probably be a long time until we manage to get to Phillip Island but we will get there & hopefully get to personally thank everyone for this amazing experience (*Facebook user*).

### Travel and conservation action

**Fig. 3:**
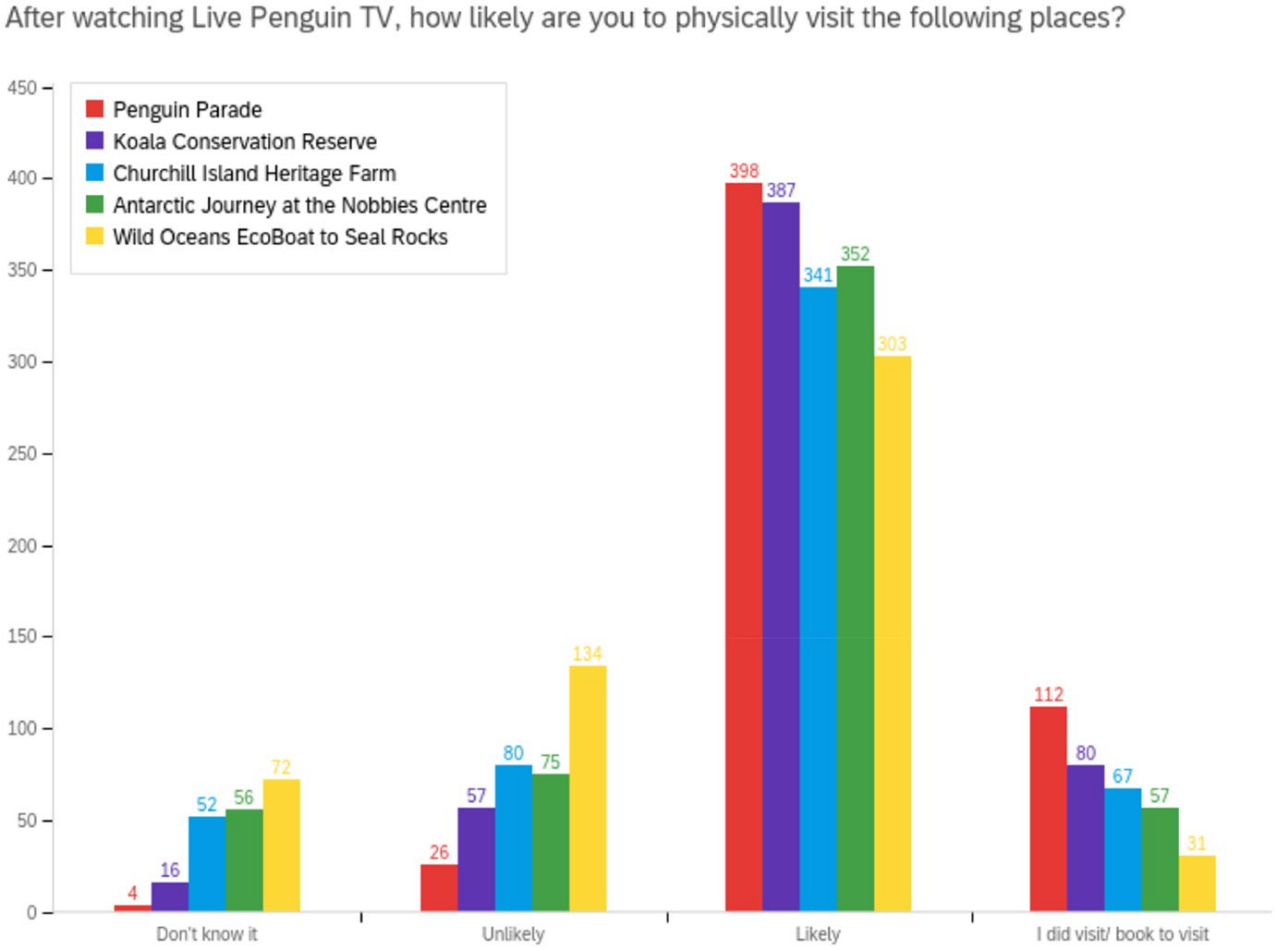
Live Penguin TV's influence on visitation to PINP’s attractions

**Fig. 4:**
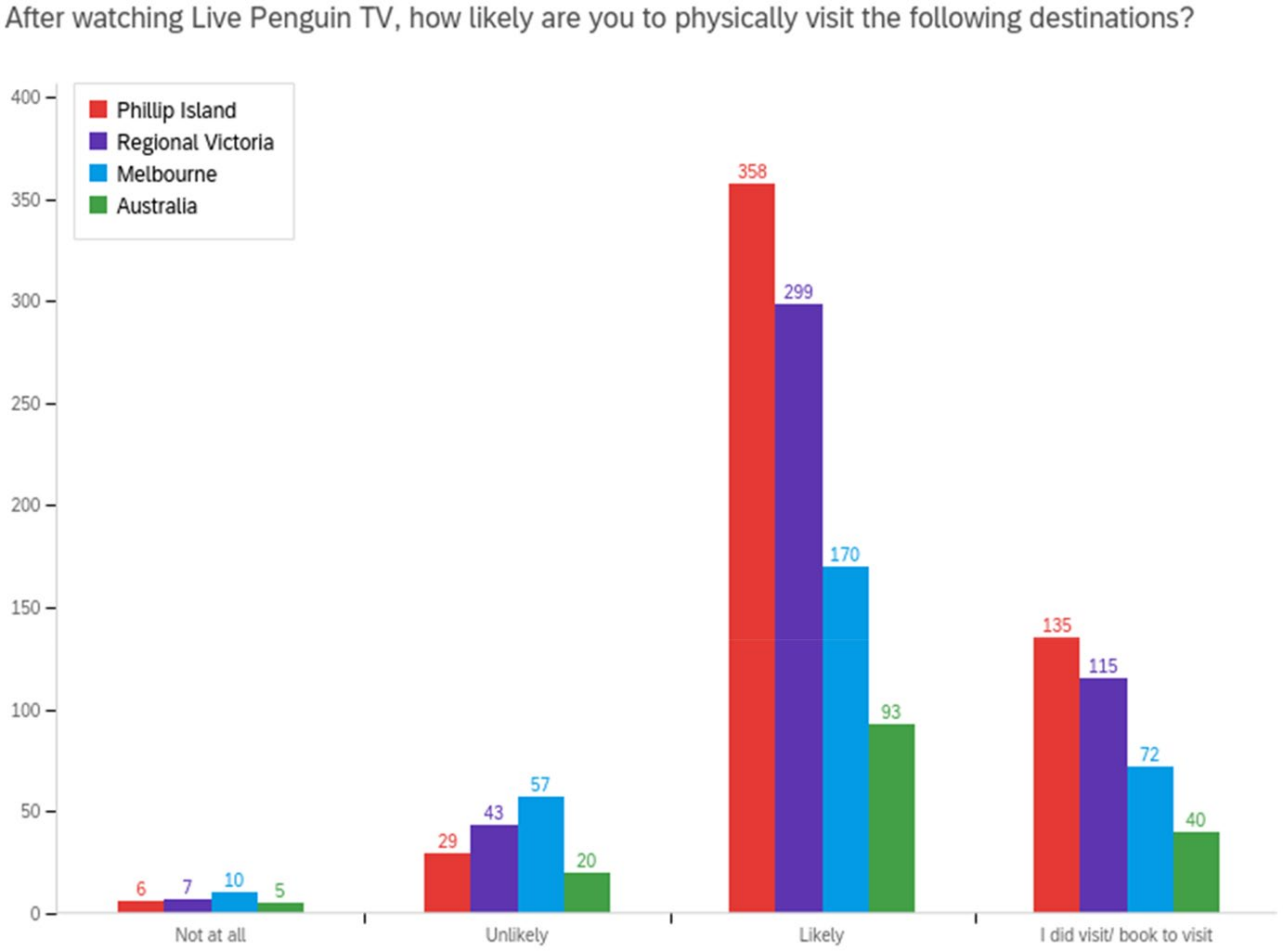
Live Penguin TV's influence on travel motivation and visitation to destinations

Increased likelihood to travel, bookings and visitation were all reported for Phillip Island and the Penguin Parade, as well as PINP’s other main sites (see Fig. 3). International, interstate and intrastate survey respondents also indicated that they were influenced to travel to Australia, Melbourne, Phillip Island and other parts of regional Victoria after watching Live Penguin TV (see Fig. 4).

Qualitative data provided specific examples of reported and planned visitation:

We went for a weekend visit to Phillip Island as soon as we could after lockdown (*Survey respondent, Melbourne, male, 30–39, watched 2–3 times weekly).*

Have already followed up with a real life viewing with the grandchildren (*Survey respondent, Goldfields in Victoria, female, 50–59, watched 5–10 times*).

My boys watch every night and are so excited that we are coming to visit you in 11 days (YouTube* user*).

We will be visiting you very soon & I hope to see a now very familiar face to say Hi to in person *(Facebook user*).

**Fig. 5:**
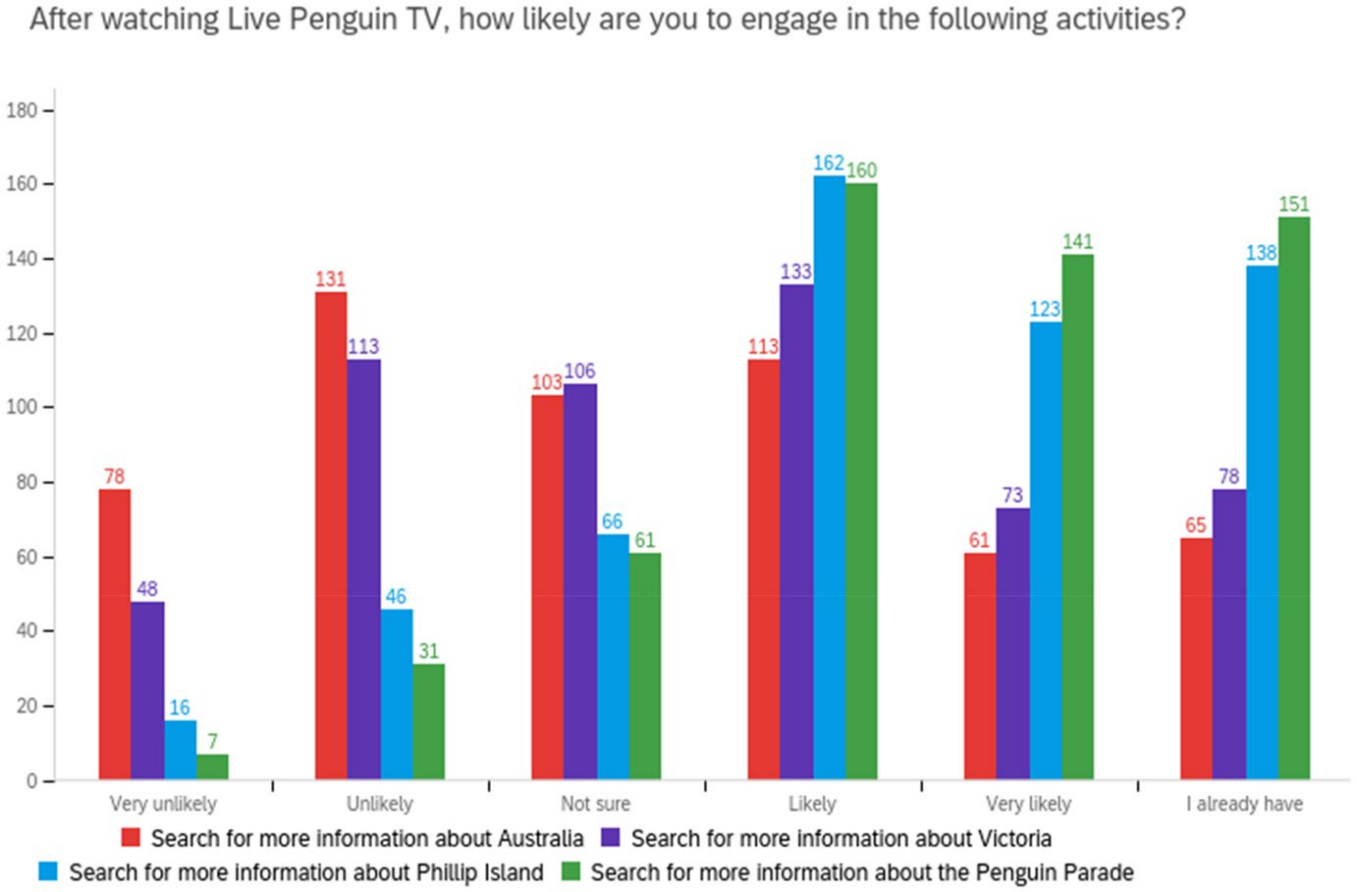
Live Penguin TV influence on information searches

Other reported actions taken included increased information searches about Australia (24% of international respondents did and 42% were likely to), Victoria (29% of international and interstate respondents did and 78% were likely to), Phillip Island (25% of all respondents did and 51% were likely to), and the Penguin Parade (27% of all respondents did and 55% were likely to), as shown in Fig. 5.

**Fig. 6:**
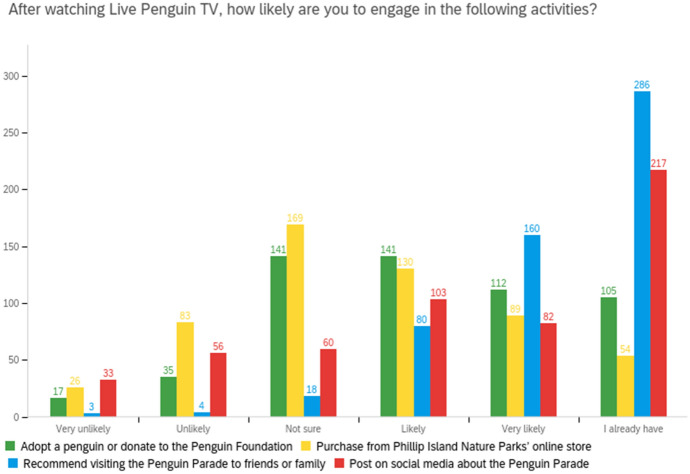
Live Penguin TV's influence on conservation action and awareness

Many viewers were influenced to take indirect conservation action with donations (19% of all respondents did and 46% were likely to) or purchasing from PINP’s online store (10% of all respondents did and 40% were likely to), or through increasing awareness by recommending friends or family visit the Penguin Parade (19% of all respondents did and 44% were likely to) or by posting about the Penguin Parade on their social media (39% of all respondents did and 35% were likely to) as shown in Fig. 6. Qualitative data provided specific examples of sustainability and conservation awareness:

Raised my awareness of conservation and clean living (*Survey respondent, Goldfields in Victoria, female, 60–69, watched daily*).

I would be not only entertained BUT educated about Penguins, Phillip Island and conservation of these treasures (*Survey respondent, Gippsland in Victoria, female, 70–79, watched 4–6 times weekly*).

It made me more interested in wildlife and conservation (*Survey respondent, Mornington Peninsula in Victoria, female, 40–49, watched 4–6 times weekly*).

The conservation message is vital for the penguins and the world at large (*Facebook user*).

Stop pollution, don't use plastic, drive less, always choose sustainable, switch your lights off! (*YouTube user*).

Thank you for always acknowledging the amazing job the Indigenous people did of managing the land and its creatures sustainably for thousands of years (*YouTube user*).

On PINP’s website, users can donate either directly to the Penguin Foundation or by symbolically adopting an animal. There was much more online commentary and questions around adopting a penguin than about donating and viewers enjoyed sharing the name they had given their penguin, suggesting that this aspect of personalisation engaged them:

Huck huck! From Wurrundjeri lands to our ‘adopted’ penguins Ngalinggu, Yawawa, and Andromezia :) (*YouTube user*).

Can’t wait to see Penelope, my adopted penguin, I’m sure she’s coming in tonight next raft (*YouTube user*).

This kept me and my daughter so captivated and happy and I adopted a penguin for her from you (*Survey respondent, UK, female, age not disclosed, watched daily).*

Thank you to everyone in front of and behind the scenes who made this all possible and who work so hard for conservation and the protection of not only the Little Penguins, but so many wonderful creatures … I will continue to share what I've learned and hope to inspire others to live as sustainably as possible. Once I am able to, I will adopt a penguin; I might already have a name picked out (*Facebook user*).

Survey respondents indicated that planned visitation to destinations and PINP’s attractions would be relatively spread out over the next five years. While some Australian state border restrictions had eased in December 2020 when the data was collected, the recency and media reports of more COVID-19 cases may have led to some hesitancy to travel within the next month.

## Conclusion

This paper has investigated an innovative campaign designed and implemented by a nature-based wildlife tourism operator as a response to the COVID-19 lockdowns and travel restrictions of 2020/21. Live Penguin TV received global media coverage and viewership numbers in the millions and succeeded in keeping PINP relevant and ‘top of mind’ in the marketplace and in the eyes of the state government, which was critical to maintain funding for conservation activities. Pre-pandemic, PINP had strong international visitation with significant markets in China and India. At the time of writing, international travel has yet to recover in Australia and this lack of tourism income presents a major threat to conservation initiatives that are dependent on tourists’ spending.

Analysis of the data reveals that Live Penguin TV positively influenced travel motivation and actual visitation, supporting the argument that viewers are more likely to visit places they had viewed through webcams (Jarratt 2020b). The findings suggest that PINP’s approach to webcam travel experiences can contribute to the post-COVID tourism recovery for Phillip Island through engaging online audiences to promote increased visitation from both domestic and international travellers. This study also shows that interactive webcam viewers actions can help to protect wildlife and their habitats through increased donations and visitation, the funds from which are directly invested into conservation work.

The only other empirical study to examine the use of webcams during the COVID-19 related travel restrictions placed focus on webcam travel user activity (Jarratt 2020b). While not explicitly stated, these all appeared to be the usual unstaffed form of webcam. Where the research in this paper departs from Jarratt’s (2020a; 2020b) work is in the observation of seemingly unique approaches to webcam livestreaming adopted by the tourism industry that do not fit the common characteristics of webcam travel that are usually said to offer no interpretation and very limited to no interaction. Through examining a scheduled, hosted and interactive model of webcam travel that provided interpretation of a specific natural event during COVID-19 lockdowns, this current study contributes a new perspective and insights into this emerging area of research on webcams in tourism.

In terms of theoretical implications, the study findings support and expand earlier research. Firstly, findings confirm that telepresence through webcam travel can provide reminiscence of pre-COVID times and a sense of connection with the outside world, especially nature and wildlife (Jarratt 2020b). Secondly, the study supports research claiming that virtual tourism experiences can instil a willingness to protect places and environments (Hofman et al. 2021). The study adds new findings that interaction, as well as elements of gamification and personalisation, can enhance webcam user engagement and connectivity through building online communities, which can also support user wellbeing.

On a practical level, the findings can be useful for industry practitioners looking to maintain and build new connections with audiences during times of crisis when physical visitation is restricted. Using Live Penguin TV as a case study, tourism businesses and stakeholders can use the learnings to inform and develop their own webcam related marketing campaigns. As a subset of virtual tourism, interactive webcam travel emerges as an alternative to more complex and/or costly forms of virtual reality. In terms of challenges associated with the interactive element of webcam travel on socia media, managers need to consider either a high level of chat moderation or clear instructions as to where/how viewers should submit questions to manage viewers’ interactive experience.

Future research is required regarding the content, timing and style of interpretation regarding interactive webcam travel as well as virtual travel experiences more broadly. While previous research has found that interpretive content did not play a significant role in motivating pro-environmental action (Hofman et al. 2021), the findings of this research suggest that the para-social relationships generated with park ranger hosts played a critical role in viewers’ experience. This study did not analyse the messages within the interpretation and commentary provided by the park ranger hosts and further investigation here would be valuable. This research also has several limitations. Firstly, the survey sample used for data collection may not be sufficiently representative of the entire population from which it was drawn due to a variety of factors, including the convenience sampling method and the unknown percentage of viewership restricted by internet censorship. Secondly, streaming software problems on social media platforms might also distort findings, for example, some viewers’ comments were not captured due to technical issues with PINP’s social media accounts and other viewers may have been unable to post comments due to connectivity issues.

To summarise, tourism destinations are particularly vulnerable to states of emergency with serious implications for the local community and conservation projects that depend on tourist spending. The success of the PINP virtual tourism experience campaign suggests that interactive webcam travel can be employed to offset at least some of the impacts caused by the lack of visitation during a health crisis or natural disaster by engaging new and old audiences online, encouraging them to donate in the short-term and to return and visit the destination/business to support recovery once it is safe to do so.

## Data Availability

The data that support the findings of this study are available from the corresponding author upon request.
